# Diagnostic Challenges in Capnocytophaga canimorsus Sepsis: A Case Report

**DOI:** 10.7759/cureus.98106

**Published:** 2025-11-29

**Authors:** Masaatsu Kuwahara, Yusuke Matsuda, Jun-ichi Hirata

**Affiliations:** 1 Department of Emergency Medicine, Takarazuka City Hospital, Takarazuka, JPN; 2 Department of Emergency and Critical Care Medicine, Hyogo College of Medicine, Nishinomiya, JPN

**Keywords:** capnocytophaga canimorsus, ct, dogs, gram-negative bacterial infection, sepsis

## Abstract

*Capnocytophaga canimorsus* infections, though rare, can be severe. Diagnosis is straightforward with a known dog-bite history, but is challenging otherwise.

A 67-year-old woman with no immunodeficiency or alcohol abuse history presented with high fever (41.5°C), vomiting, and altered consciousness. No neck stiffness or peritoneal irritation was observed. Laboratory tests showed elevated liver enzymes, creatine kinase, inflammatory markers, and lactate. Computed tomography suggested strangulated ileus, which was ruled out during emergency laparotomy. Postoperatively, she experienced shock and was treated with vasopressors, corticosteroids, and ampicillin-sulbactam. Her condition improved, and blood culture showed Gram-negative rods. On day 3, her husband mentioned a recent dog bite, and a *Capnocytophaga *infection was suspected. Continued treatment with ampicillin-sulbactam improved her symptoms, and she was discharged. Blood cultures confirmed *C. canimorsus* on day 10.

In severe sepsis with delayed bacterial identification, *C. canimorsus* should be considered. Repeated history-taking and empirical beta-lactamase inhibitor treatment are crucial.

## Introduction

Although rare, the *Capnocytophaga canimorsus *bacterium can cause severe infections in humans [1]. The Dutch report indicated an incidence rate of 0.67 cases per million population [2]. This often occurs after dog bites; however, its diagnosis becomes challenging if a history of dog bites is unclear for patients [3]. Herein, we report a case of *C. canimorsus *sepsis in a patient with an initially unknown dog-bite history, complicating its differentiation from meningitis and strangulated ileus.

## Case presentation

The patient was a 67-year-old female with no history of immunodeficiency, medication use, or heavy alcohol use. She experienced fever and vomiting beginning the day before her admission to the hospital. On the day of her admission, she developed a fever and altered consciousness, prompting emergency medical attention.

Upon arrival, her temperature was 41.5°C, with a Glasgow Coma Scale score of 4-2-5, indicating a confused state. Her blood pressure was 128/74 mmHg, pulse was 96 bpm, respiratory rate was 30/min, and her SpO_2_ was 97% on 10 L of oxygen. Physical examination was limited, owing to agitation; however, no signs of neck stiffness or peritoneal irritation were noted. Her abdomen was mildly distended without marked tenderness, and her breath sounds were clear bilaterally. There were no obvious skin lesions or signs of a bite. Initial investigations included blood tests, urinalysis, whole-body computed tomography (CT), blood cultures, and lumbar puncture. Blood tests revealed mildly elevated liver enzymes, creatine kinase, inflammatory markers, and lactate levels (Table 1).

**Table 1:** Laboratory findings on admission Lac: lactate; WBC: white blood cell; Hb: hemoglobin; PLT: platelet count; PT: prothrombin time; TP: total protein; ALB: albumin; T-Bil: total bilirubin; AST: aspartate aminotransferase; ALT: alanine aminotransferase; BUN: blood urea nitrogen; Cre: creatinine; CK: creatine kinase; CRP: C-reactive protein; Glu: glucose; Na: sodium; K: potassium; Cl: chloride; Ca: calcium; Mg: magnesium

Test	Results	Units	Normal Range
Lac	60	mg/dL	4.5-14.4
WBC	2630	μ/L	3300-8600
Hb	12.5	g/dL	13.7-16.8
PLT	89000	μ/L	15800-34800
PT	55	%	70-130
TP	5.6	g/dL	6.6-8.1
ALB	3.3	g/dL	4.1-5.1
T-Bil	2.3	mg/dL	0.4-1.5
AST	55	U/L	13-30
ALT	30	U/L	10-42
BUN	14.5	mg/dL	8.0-20.0
Cre	0.81	mg/dL	0.65-1.07
CK	535	U/L	59-248
CRP	10.06	mg/dL	<0.14
Glu	124	mg/dL	73-109
Na	133	mmol/L	138-145
K	3.3	mmol/L	3.6-4.8
Cl	98	mmol/L	101-108
Ca	8.6	mg/dL	8.8-10.1
Mg	1.5	mg/dL	1.6-2.6

**Figure 1:**
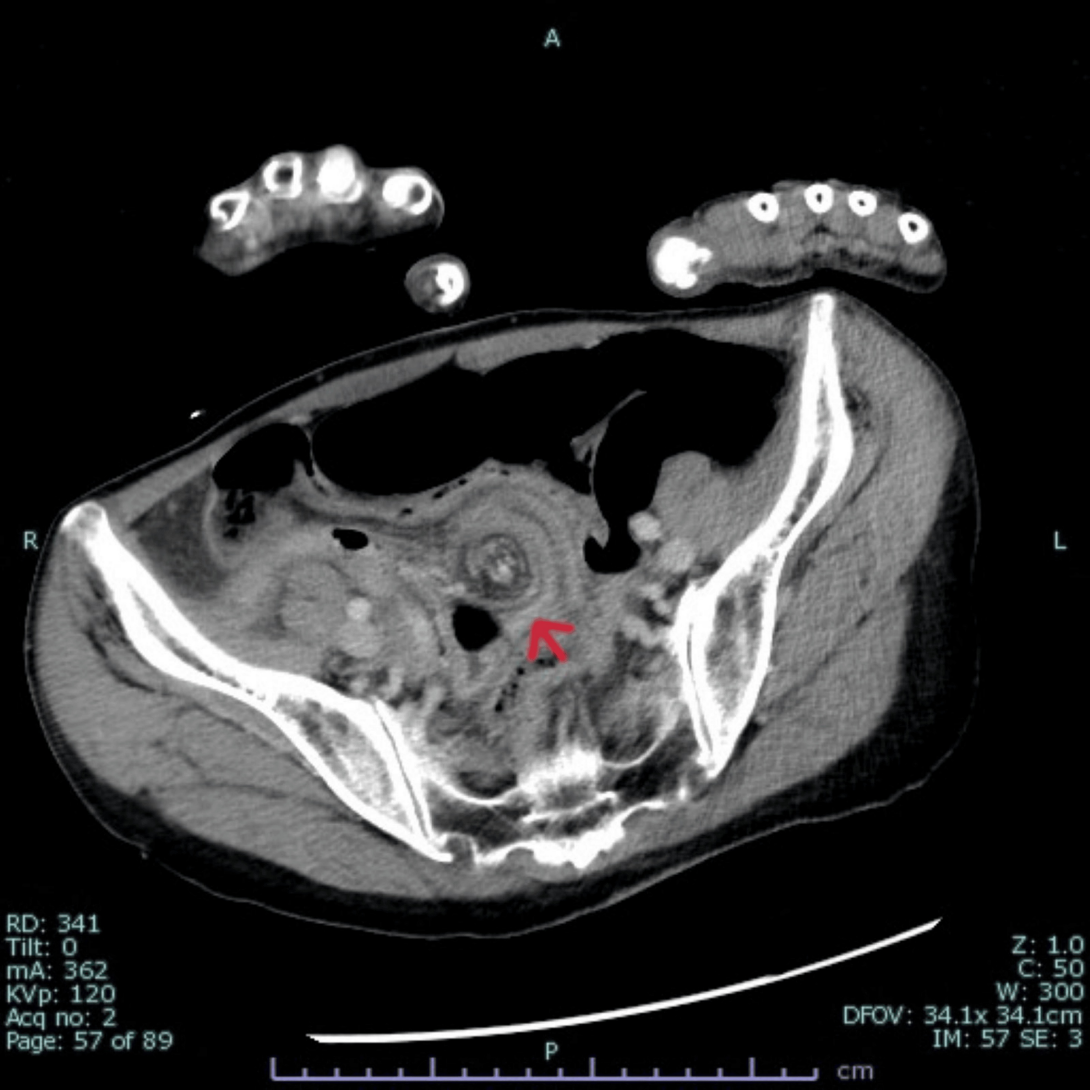
Contrast-enhanced abdominal CT on admission The arrow indicates the suspected site of mesenteric torsion.

CT revealed a whirlpool sign in the abdomen and small bowel distension (Figure 1). Cerebrospinal fluid analysis did not reveal any signs of meningitis.

Based on our CT findings, the presence of mild abdominal distension, the patient’s history of vomiting, and her elevated lactate levels, we suspected volvulus that had led to strangulated ileus with bacterial translocation, ultimately causing sepsis. Empirical antibiotics (ampicillin-sulbactam, 3 g) were administered, and an emergency laparotomy was performed. A volvulus was noted intraoperatively, but bowel perfusion was preserved, thus ruling out the possibility of strangulated ileus. Bowel resection was not required, and the volvulus was corrected.

Postoperatively, the patient was admitted to the intensive care unit (ICU) but developed shock with a systolic blood pressure of 50 mmHg. Vasopressors (norepinephrine and vasopressin) and corticosteroids (200 mg/day) were administered. Her consciousness gradually improved; however, her dog-bite history remained unknown.

On the second day, Gram-negative rods were reported in the patient’s blood cultures, but the specific species had not yet been identified. On the third day, her husband mentioned that she had been bitten on the right arm by a dog five days before her admission. The patient confirmed this finding by stating that she had self-treated the wound at home. The bite site was initially red and swollen, but had resolved by the time she was admitted, with only faint marks remaining.

Given the slow-growing Gram-negative rods in her blood cultures and the patient’s dog-bite history, we suspected *Capnocytophaga* infection. Despite the culture results still being pending, we continued the ampicillin-sulbactam treatment. The patient’s condition improved; she was weaned off catecholamines on day 4 and transferred out of the ICU. The patient ultimately completed a 10-day course of ampicillin-sulbactam treatment.

Blood cultures confirmed the presence of *C. canimorsus* on day 10. The patient underwent rehabilitation and was discharged home on day 15.

## Discussion

Herein, we report a challenging case of *C. canimorsus* sepsis. The diagnosis was complicated by several factors, as listed below:

Severe altered consciousness

The patient had a significantly altered consciousness upon admission, which hindered detailed history-taking. Her husband was away on a business trip, which further limited the availability of information. *C. canimorsus* sepsis is known to cause severe impairment of consciousness [4-6], which complicates its diagnosis when the patient’s history is unclear.

CT findings

The whirlpool sign on our patient’s CT suggested volvulus, complicating the differentiation of her diagnosis from bacterial translocation-induced sepsis. Recent reports have indicated that whirlpool signs can be asymptomatic [7]. In this case, the volvulus did not cause ischemia or require surgical intervention, highlighting the need for additional case studies.

Absence of immunocompromising conditions

The patient had no history of splenectomy, liver cirrhosis, or excessive alcohol consumption, which are all common in cases of *Capnocytophaga* infection [8]. This atypical history further complicated the diagnosis.

Despite the diagnostic challenges, appropriate antibiotic selection was critical for patient survival in this case. On the second day of the patient’s admission, Gram-negative rods were detected in her blood cultures. These are typically identifiable by species by the third day; however, this identification was delayed, and the patient’s dog-bite history was revealed in the meantime. *Capnocytophaga* species exhibit slow culture growth and beta-lactamase production [9]. Ampicillin-sulbactam was continued to effectively treat the infection.

Patients with severe infections should be treated with beta-lactam-beta-lactamase inhibitor combinations (e.g., ampicillin-sulbactam, piperacillin-tazobactam) or carbapenems (e.g., imipenem, meropenem) while awaiting susceptibility test results. Ampicillin-sulbactam (3 g intravenously every six hours) is recommended over piperacillin-tazobactam or carbapenems due to its narrower antimicrobial spectrum. As an exception, carbapenems are recommended for patients with meningitis caused by the genus *Capnocytophaga*, as the combination of a beta-lactam and a beta-lactamase inhibitor does not achieve sufficiently high concentrations in the cerebrospinal fluid.

Less severe infections may be treatable with oral therapy (e.g., amoxicillin-clavulanate 875 mg orally every 12 hours). Clindamycin is an alternative, but due to the risk of *Clostridium difficile* infection with clindamycin, a beta-lactam-beta-lactamase inhibitor is recommended.

The identified *C. canimorsus* strain produced the class D beta-lactamase blaOXA-34, emphasizing the importance of beta-lactamase inhibitor antibiotics in such cases, until susceptibility results are available.

## Conclusions

*C. canimorsus* infection should be considered in cases of severe sepsis with delayed bacterial identification, even in the absence of typical risk factors such as immunocompromise or evident bite marks. This case highlights that repeated and meticulous history-taking can uncover critical exposure information that directly guides antimicrobial decision-making. Empirical use of beta-lactamase inhibitor antibiotics remains essential until pathogen identification and susceptibility results are available, particularly because *Capnocytophaga* species may produce clinically relevant beta-lactamases.
